# The effect of desert dust particles on rheological properties of saliva and mucus

**DOI:** 10.1007/s11356-019-04628-x

**Published:** 2019-03-04

**Authors:** Agata Penconek, Urszula Michalczuk, Agnieszka Sienkiewicz, Arkadiusz Moskal

**Affiliations:** Faculty of Chemical and Process Engineering, Warsaw University of Technology, Waryńskiego 1, 00-645 Warsaw, Poland

**Keywords:** Desert dust, Rheology, Saliva, Mucus

## Abstract

Transported desert dust particles (TDDP) are soil particles suspended in the air. Being spread all over the globe by the winds, TDDP affect animals, including humans, plants and other organisms not only in the areas of their emission. In humans, TDDP are responsible for diseases of the respiratory (e.g. asthma) and circulatory (e.g. heart failure) systems and they also act directly on the epithelium and its mucus layer after deposition in the mouth and respiratory system. The aim of the study was to determine the influence of TDDP on the rheology of mucus and saliva, and thus on their functioning. The artificial mucus and saliva, as well as Arizona TDDP, were used in experiments. The rheological properties of TDDP were determined with the use of an oscillatory rheometer, at various temperatures and in the presence of different amount of TDDP. Moreover, the diffusion time of the marker (rhodamine B) throughout mucus with desert dust particles was examined. The obtained results demonstrate that the presence of TDDP in the saliva and mucus model increases their apparent viscosity. The concentration of particles is positively correlated with the increase of viscosity. However, it has not been demonstrated that the presence of TDDP in mucus significantly influenced the diffusion of a fluorescent marker throughout the mucus. The presence of TDDP in the saliva and mucus may interfere with their moisturising function, and cause difficulties in swallowing by increasing the viscosity of mucus and saliva. Moreover, increased viscosity of mucus may cause problems with its ability to pass to the upper respiratory tracts, which may lead to a general discomfort or local inflammation.

## Introduction

Transported desert dust particles (TDDP) are soil particles suspended in the atmosphere that come from desert and semi-desert areas. The main sources of TDDP are located in the Northern Hemisphere (Sahara and Sahel in North Africa, Southwest Asia, East Asia and the Middle East). Among them, the largest source of desert dust is the Sahara area with an estimated dust emission of 670 Mt per year (Choobari et al. 2014). TDDP are a significant atmospheric aerosol component, and therefore have a great impact upon the environment and organisms (Choobari et al. 2014). On the one hand, TDDP provide micronutrients to the natural water reservoirs and ecosystem but on the other, TDDP can interact with liquids and clouds, affect the precipitation process, absorb solar and infrared radiation and lead to undesirable atmospheric changes (Choobari et al. 2014; Mahowald et al. 2014). Moreover, TDDP suspended in the air can be a threat to animals, including human health, due to their respirable sizes. TDDP are spread far beyond their emission sources and even circle the Earth (Uno et al. 2009).

The threat posed by desert dust results not only from its size but also its composition. However, the composition of TDDP is strongly affected by the size (due to the available surface area and the degree of adsorption associated with it, which depends on the nature of the functional groups and the length of the side chains of the adsorptive chemicals) and source of dust. For example, TDDP from Australia have a higher Fe/Al ratio than TDDP from another part of the Northern Hemisphere (Radhi et al. 2010). Generally, TDDP consist of aluminium, silicon, iron, magnesium, calcium and other components like, for example, lead, sodium or potassium in various proportions depending on the place where they are generated and their size. Formenti et al. (2011) determined the main chemical components of mineral dust aerosol from Sahara as Al, SO_4_^2−^, Ca and Mg for particles with the diameter < 1 μm and Si, Al, Fe and Ca for particles with the diameter > 1 μm. The TDDP size distribution varies from 0.3 μm even up to 20 μm depending on the source of TDDP and techniques used to determine it (Mahowald et al. 2014).

Due to their size, a lot of TDDP are easily inhaled (particles with diameter below 5 μm are defined as respirable fraction) and can even penetrate the deepest region of respiratory tract—bronchioles and alveoli. Once deposited in the alveoli, they can easily enter the bloodstream (Semmler et al. 2004). Therefore, the influence of TDDP on human health is mainly considered in the context of respiratory and circulatory system diseases. The main respiratory disorders are asthma, pneumonia and tracheitis and the circulatory system diseases include myocardial infarction, stroke, heart failure, arrhythmias and venous thromboembolism (Goudie 2014; Zhang et al. 2016). The local effects of TDDP on the human body are also significant. TDDP, after their deposition, influence directly on epithelium and its mucus layer. Moreover, large particles which deposited in the upper respiratory tract are present also in the oral cavity where they interact on saliva.

Therefore, the aim of the study was to investigate the effect of the presence of TDDP in mucus and saliva on their rheology and, as a consequence, on their functions connected with flow properties.

The primary function of mucus is to protect epithelium against inhaled pathogens. The main role of saliva is to begin the process of enzymatic degradation of nutrients. But saliva also protects and lubricates the soft and hard tissues in the oral cavity against mechanical, chemical and thermal irritation. The protection and lubrication functions of mucus and saliva are strictly connected to their rheological properties, which are determined by their chemical composition, physical parameters like temperature, or pH, but also by health, age, sex or activity. From the rheological point of view, mucus and saliva are non-Newtonian, pseudoplastic fluid. The main reason of pseudoplastic behaviour of both fluids is the presence of mucins—large glycoproteins giving mucus and saliva their viscoelastic character. Generally, saliva consists in 99% (*w*/*w*) of water, and the remainder of the constituents (1% *w*/*w*) include proteins (enzymes, antibodies, mucins, etc.), electrolytes (sodium, potassium, chloride, bicarbonate), sugars and nitrogen compounds (de Almeida et al. 2008). The apparent viscosity of saliva is between 5 and 25 mPa·s (Christersson et al. 2000) and the value decreases with pH lowering (Brujan 2011). Briedis et al. (1980) showed that the type of food and emotional stress also affect the viscosity of saliva. Therefore, it is advisable to use substitutes of saliva when we are looking for the cause-effect (factor-reaction) relationship.

Several commercial substitutes of saliva are available on the market (e.g. Mucinox [Medac GmbH Sp. o.o.], Saliva rex [PATER Laboratory] and at least two laboratory models of saliva can be found in the literature (both described in Christersson et al. 2000)). Both models have identical composition but different proportions of ingredients.

The influence of dietary factors on saliva viscosity (Briedis et al. 1980), as well as the relationship between saliva viscosity and the severity of caries (Yas and Radhi 2013) or age or physical activity (Zussman et al. 2007), has been determined. However, according to the authors’ knowledge, the effect of desert dust on the rheology of saliva and thus its functions have not been determined yet.

Mucus, like saliva, consists mainly of water (93%), lipids (5%) and proteins (1%); the rest is attributed to mineral compounds. Mucins represent 15% of mucus protein (Schenkels et al., 1995). The apparent viscosity of nasal/bronchial mucosa is in the range of 0.01–1000 mPa·s (Lai et al., 2009). The apparent viscosity of mucus decreases while pH is increasing (Bansil and Turner 2006). The viscosity of mucus also decreases due to the presence of acidic pollutants (Holma and Hegg 1989), diesel exhaust particles (Penconek and Moskal 2016) or smoking (Kollerstrom et al. 1977), but increases when immunoglobulins appear in it (Girod et al. 1992). The viscoelastic properties of mucus, as well as chemical composition, are dependent on place of secretion, age, sex, physical activity or even atmospheric conditions. Therefore, also in this case, the mucus model should be used rather than native mucus, to study the influence of various factors on mucus rheology. There are several models of artificial mucus, some of them are very rich in ingredients (e.g. D'Angelo et al. 2015) and some have only two or three components (Dawson et al. 2004; McGill and Smith 2010). All mucus models have been widely employed in permeability and rheological studies.

In the study, both saliva and mucus models were used to investigate the effect of TDDP presence on their rheology. On this basis, the potential impact of TDDP on the functions of saliva and mucus was estimated. According to the authors’ knowledge, no study of this kind has taken place beforehand.

## Materials and methods

### TDDP

As an example of desert dust, the Arizona Dust (nominal 0–3 μm, Powder Technology INC, USA) was used. The Arizona Dust is commonly used in many applications such as water filter testing and air filter performance testing. It is obtained in the four-step process from raw dust from Salt River Valley (Arizona). Therefore, we assumed that Arizona Dust might be a good equivalent of natural dust.

The chemical composition of TDDP (Arizona Dust) based on the safety data sheet is shown in Table 1.

**Table 1:** The concentration of the inorganic component of desert dust particles

Component	Amount, %
Quartz	68.0–76.0
Aluminium oxide	10.0–15.0
Diiron oxide	2.0–5.0
Calcium oxide	2.0–5.0
Potassium chloride	2.0–5.0
Disodium oxide	2.0–4.0
Magnesium oxide	1.0–2.0
Titanium dioxide	0.5–1.0

The size distribution of the Arizona Dust in the air was determined with the use of spectrometer (Grimm Model 1.109, Germany). The Arizona Dust was resuspended in the tank where negative pressure was generated by the air flowing through the tank. The particle size distribution was determined by placing a meter probe inside the tank. The size distribution in water was obtained using the Zetasizer (Malvern, UK).

The morphology of Arizona Dust was described based on SEM images (Zeiss Ultra Plus, Germany).

### Body fluids

We use artificial saliva and artificial mucus because too many factors influence natural mucus and saliva composition and, therefore, their rheological properties.

#### Saliva

Artificial saliva was obtained based on the model described in Christersson et al. (2000). The benzalkonium chlorides ((Sigma Aldrich) [0.02 g/l], EDTA (Sigma Aldrich) [0.5 g/l], NaF (Chempur) [0.0042 g/l], xylitol (Sigma Aldrich) [20 g/l], methylparaben (Sigma Aldrich) [1 g/l], mucins (type II) (Sigma Aldrich) [35 g/l]) were dissolved in deionised water and then placed on a magnetic stirrer (500 rpm) for 2 h. The pH of the solution (7.00) was determined using NaOH or HCl. The sample was stored and sealed at 4 °C.

#### Mucus

At the first stage of the research, three mucus models (D'Angelo et al. 2015; Dawson et al. 2004; McGill and Smith 2010) were created in the laboratory, and their stability over time and the impact of individual components were determined. All three models exhibited similar stability over time and similar rheological properties in changing pH and temperature changing. The presence of stabilising substances, a substance that reduce surface tension and presence of ions, does not significantly affect the properties of the investigated artificial mucus if only rheological properties are taken into account. Therefore, because the aim of the research is to determine changes in fluid rheology as a result of the presence of desert dust, it was decided to use the simplest model (McGill and Smith 2010) for further research.

This model was also used in our previous work to determine the impact of diesel exhaust particles on mucus rheology (Penconek and Moskal 2016).

The mucins type II (Sigma Aldrich) (the concentration of mucins in models 1 and 2 was 20 g/l and 200 g/l respectively) and NaN_3_ (POCH, Poland) (0.01 g/l) were dissolved in deionised water and then placed on a magnetic stirrer (500 rpm) for 2 h. After this time, the pH of the solution (7.4) was adjusted with HCl or NaOH. The mucus was stored in a closed chamber at 4 °C.

### Methodology

#### Rheological properties

We investigated the rheological properties of saliva and mucus with TDDP. The Arizona Dust was added to saliva or mucus (prepared the day before measurement) in the concentrations of 0.06 g/l and 6 g/l. After that, the TDDP suspension was stirred for 15 min. The saliva and mucus were warmed in a water bath to room temperature before adding the desert dust. The rheological properties (flow curve and the dependence of viscosity as a function of shear stress) were examined with oscillation rheometer (MCR102, Anton Paar, Austria) equipped with a Peltier system in a plate-plate system for a 1-mm-wide gap. The tests were carried out at three temperatures: 22 °C, 36.6 °C and 40 °C.

#### Protective properties of mucus

We also studied the influence of the presence of TDDP in the mucus on its protective properties. We assumed that the diffusion time of fluorescent marker through mucus could be a good indicator of changes in mucus protective properties.

**Fig. 1:**
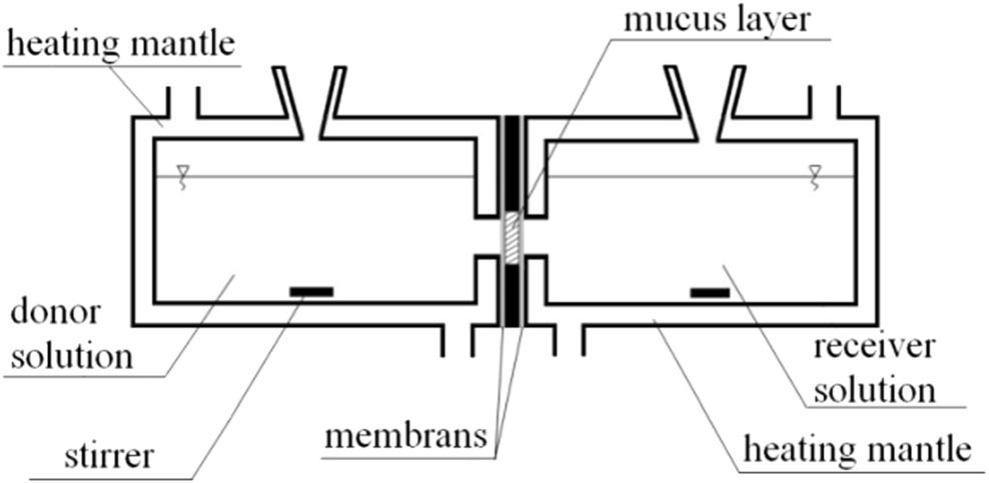
Diffusion cell

The measurement of time of diffusion was conducted in a side-by-side horizontal cell (PermeGear, USA). The mucus was placed between membranes (pore diameter 100 nm, thickness 100 μm) (Durapore, Millipore, Ireland) as shown in Fig. 1. The thickness of the mucus layer was 1.5 mm, and its surface area was 19.6 mm^2^. The donor compartment was filled with the solution of fluorescent marker—rhodamine B (Sigma Aldrich) (0.5 g/l), and the acceptor compartment was filled with the RO water (PURICOM). The donor and acceptor solution were stirred throughout the test to reduce the local mass transfer resistance. The tests were conducted at body temperature (36.6 °C).

The concentration of marker in the acceptor side was determined spectrofluorimetrically (Lumina Fluorescence Spectrometer, Thermo Scientific, USA) at an excitation wavelength 554 nm and emission wavelength 578 nm. The fluorescence spectrum was recorded in the wavelength range from 500 to 700 nm. The concentration of rhodamine B in acceptor solution was determined from the standard curve prepared beforehand. Samples were taken from the acceptor compartment at the 30-min intervals. The results are presented as the average value from at least three tests.

## Results and discussion

### Desert dust

**Fig. 2:**
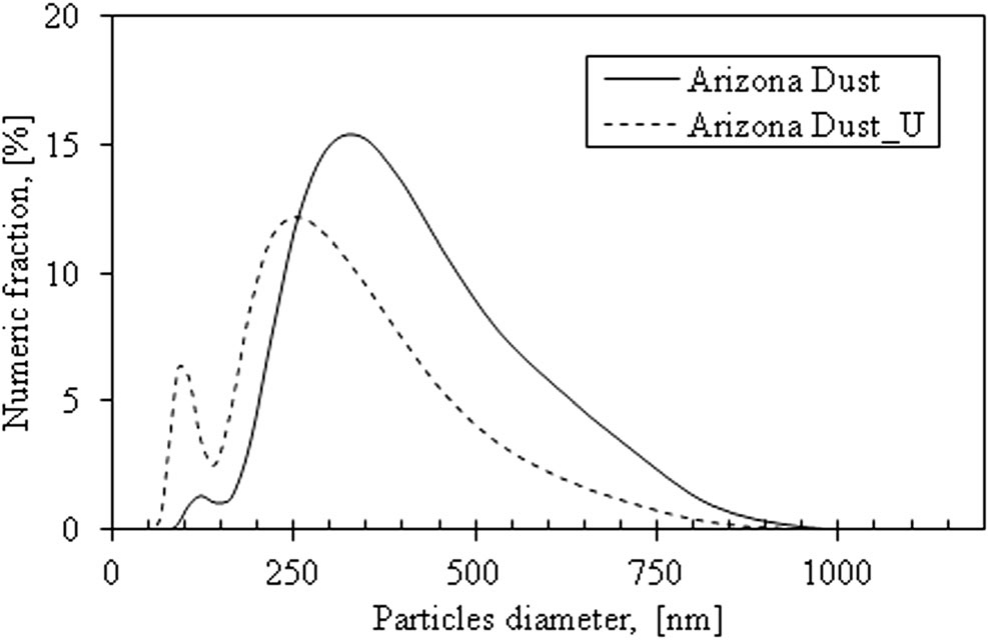
The TDDP size distribution before (solid line) and after sonification (dotted line)

The average diameter of TDDP in the air is 1.968 μm, while in water, the TDDP diameter is 0.513 μm (the particles were measured in a size range from 0.18 to 1 μm). The average size of TDDP after sonification in water is 433 nm (Fig. 2).

**Fig. 3:**
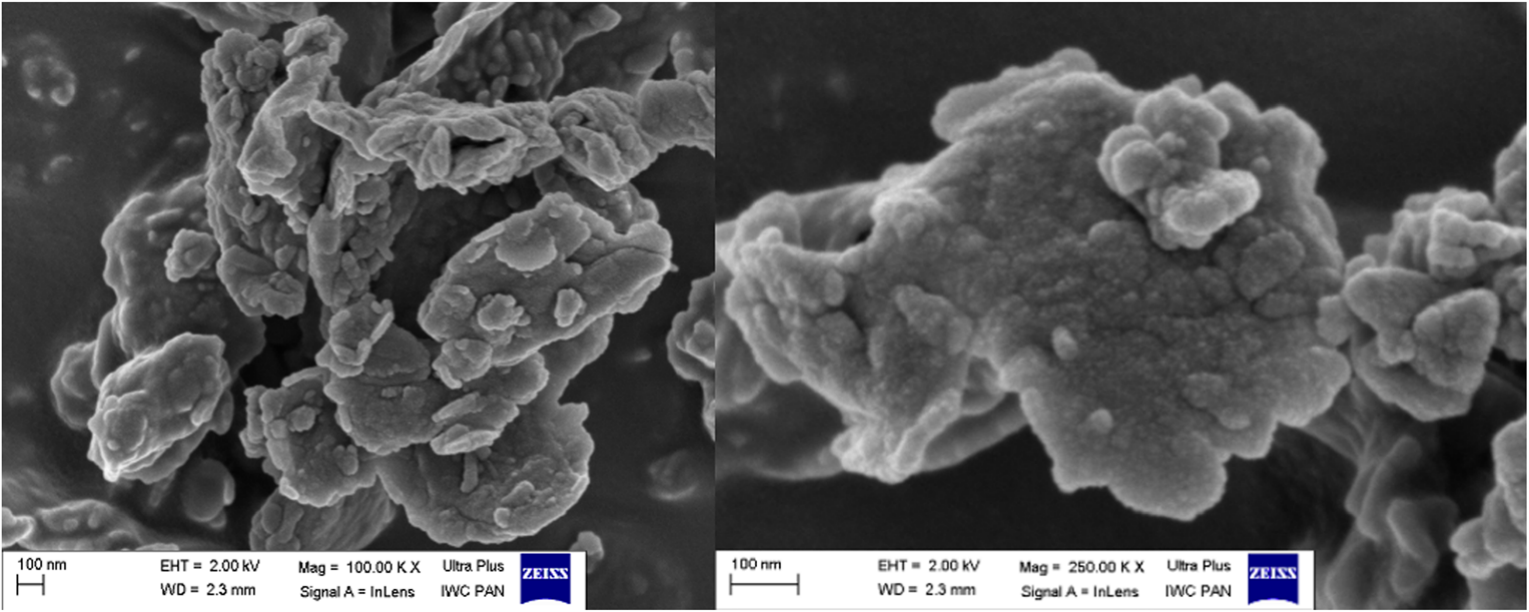
The aggregates of desert dust particles

The TDDP tend to form aggregates in the air (Fig. 3) which explains their higher average diameter TDDP in the air than in water. The average diameter in the water is almost four times lower than in the air, and it does not significantly change after sonication. It may suggest that TDDP disaggregate in water, but after this process, the obtained forms of particles are rather stable.

### The influence of desert dust on saliva rheology

**Fig. 4:**
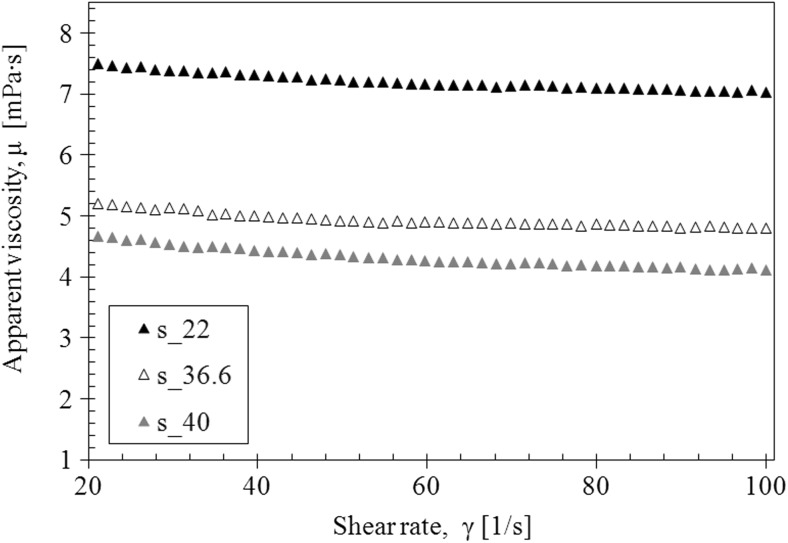
The apparent viscosity of saliva model

**Fig. 5:**
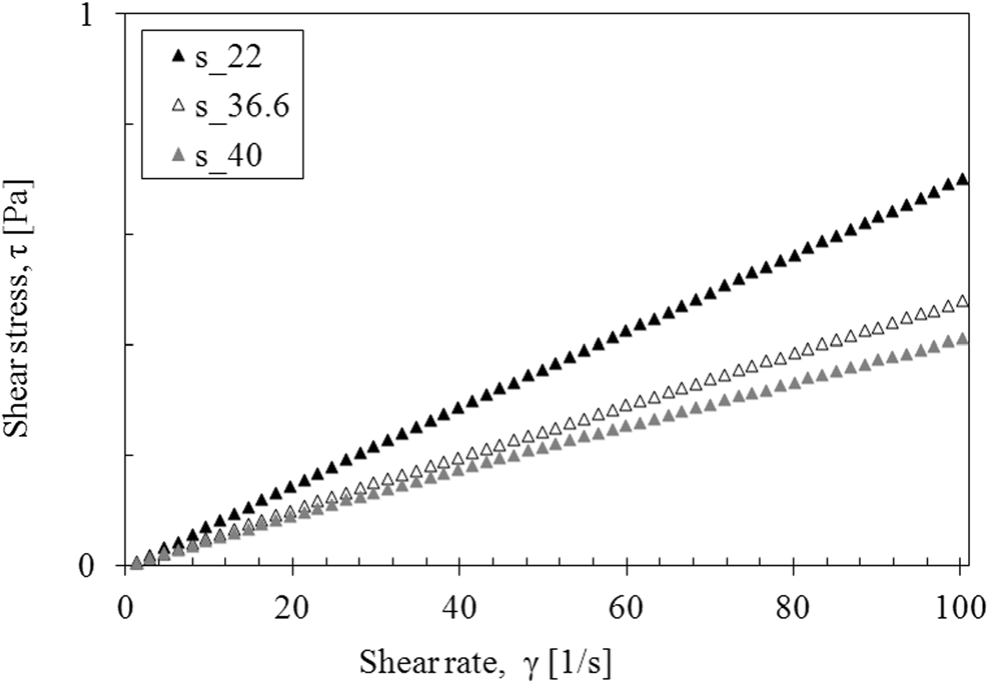
The flow curves of saliva model

The saliva model behaves slightly like a pseudoplastic fluid—the viscosity decreases with the increase of the shear rate at all examined temperatures (Fig. 4). However, the flow curves are straight line, which is a characteristic of Newtonian fluids (Fig. 5).

**Fig. 6:**
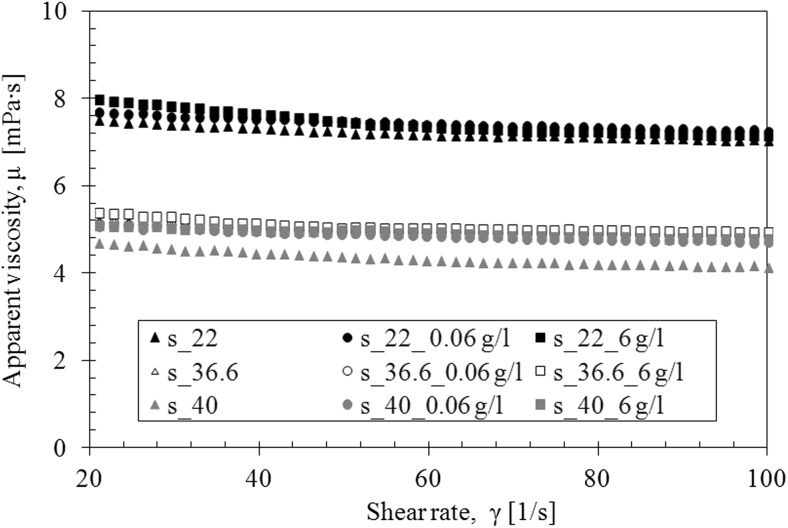
The influence of TDDP on apparent viscosity of saliva model

**Fig. 7:**
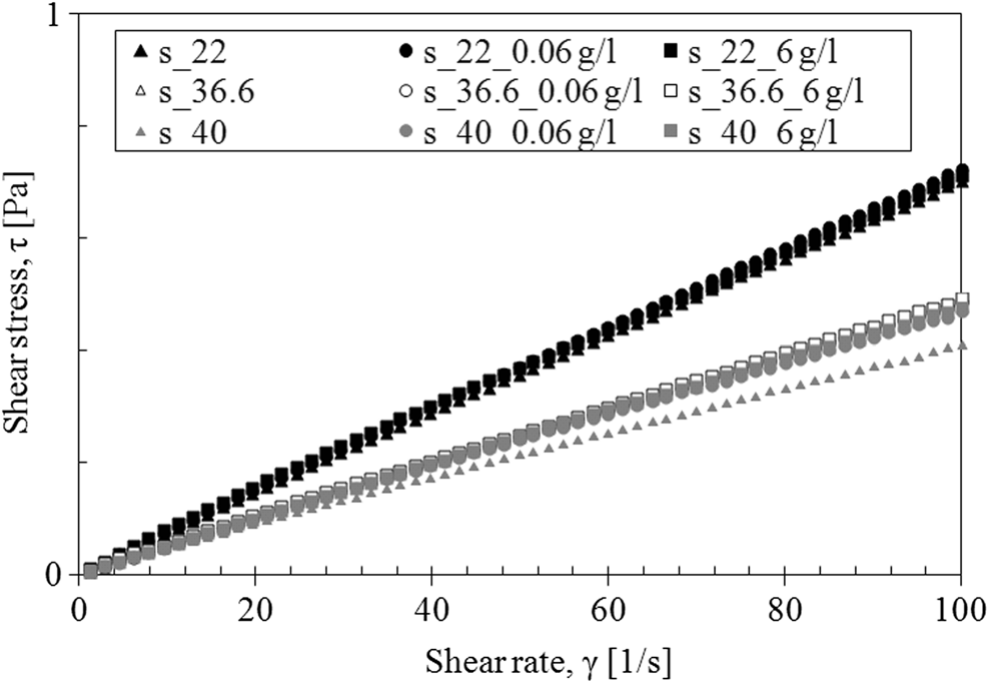
The influence of TDDP on flow curves of saliva model

The presence of TDDP in laboratory saliva slightly increased the viscosity (Fig. 6). The influence of TDDP on saliva viscosity is the most visible at the temperature of 40 °C. Moreover, the addition of particles does not change the shape of the flow curves—regardless of particles’ concentration (Fig. 7).

Biesbrock et al. (1992) and Yas and Radhi (2013) found that there are relationships between the viscosity of saliva and the extent and incidence of dental caries and periodontal disease. While the salivary viscosity increases, dental caries also increases. They did not define if dental caries increases because the viscosity of saliva increases or the viscosity increases because the dental caries increases. However, the relationship between salivary viscosity and dental caries is sufficient to assume that an increase in the viscosity of saliva due to the presence of TDDP may have adverse health consequences.

Moreover, while the saliva viscosity increases, the bacteria co-aggregation decreases that leads to disruption in oral clearance and, as a consequence, may even cause the increases in the likelihood of aspiration pneumonia and cardiovascular diseases especially in the elderly (Kitada and Oho 2011).

### The influence of desert dust on mucus rheology

The rheological properties of the two mucus models are different due to the various amounts of mucins.

**Fig. 8:**
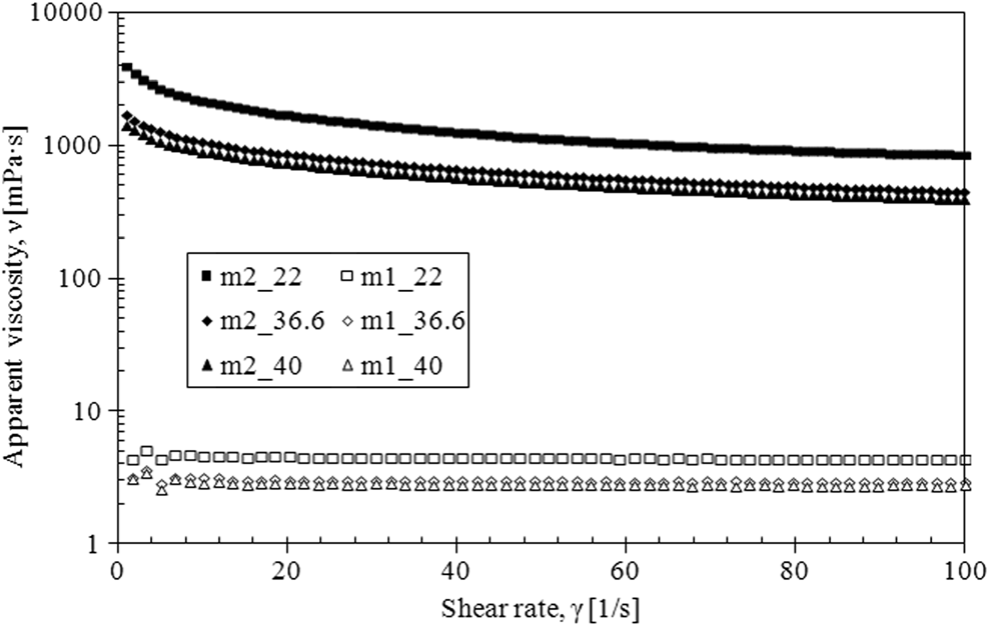
The apparent viscosity of mucus model (m1, mucus model 1; m2, mucus model 2)

The mucus model 1 behaves like Newtonian fluid—the viscosity is constant upon increasing shear rate for all investigated temperature while the mucus model 2 behaves like a pseudoplastic fluid—the viscosity decreases upon increasing shear rate for all investigated temperature (Fig. 8).

**Fig. 9:**
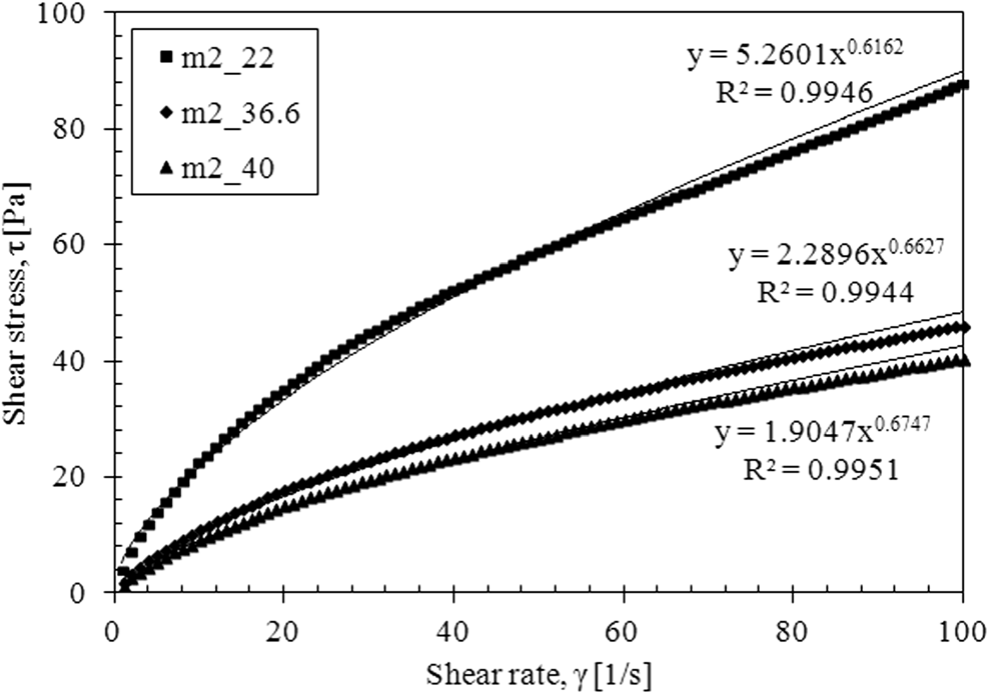
The flow curves for mucus model 2

**Fig. 10:**
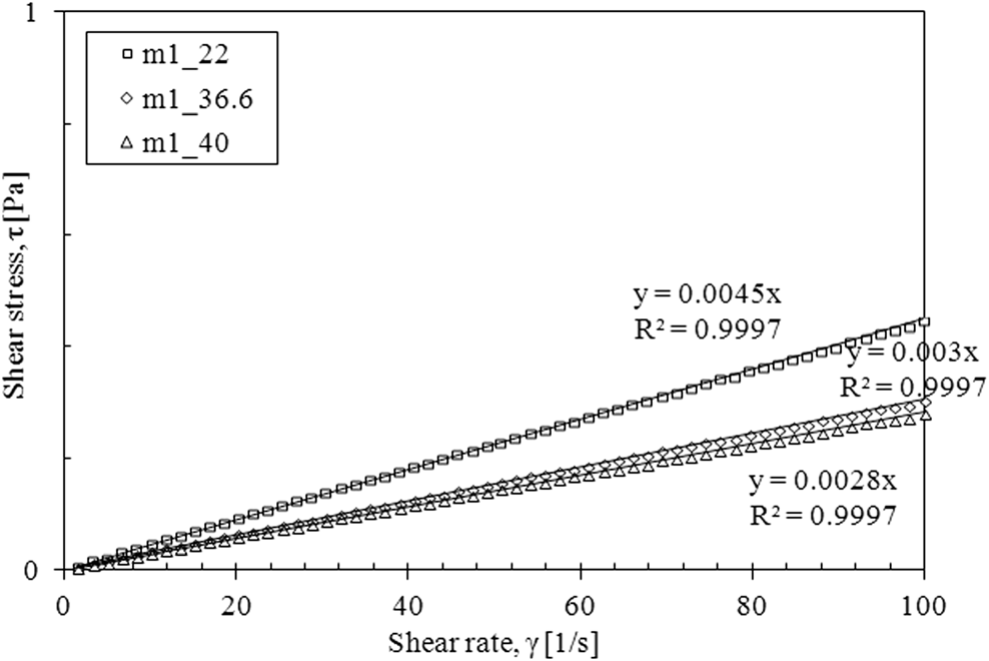
The flow curves for mucus model 1

The flow curves for both models confirm these observations (Figs. 9 and 10).

**Fig. 11:**
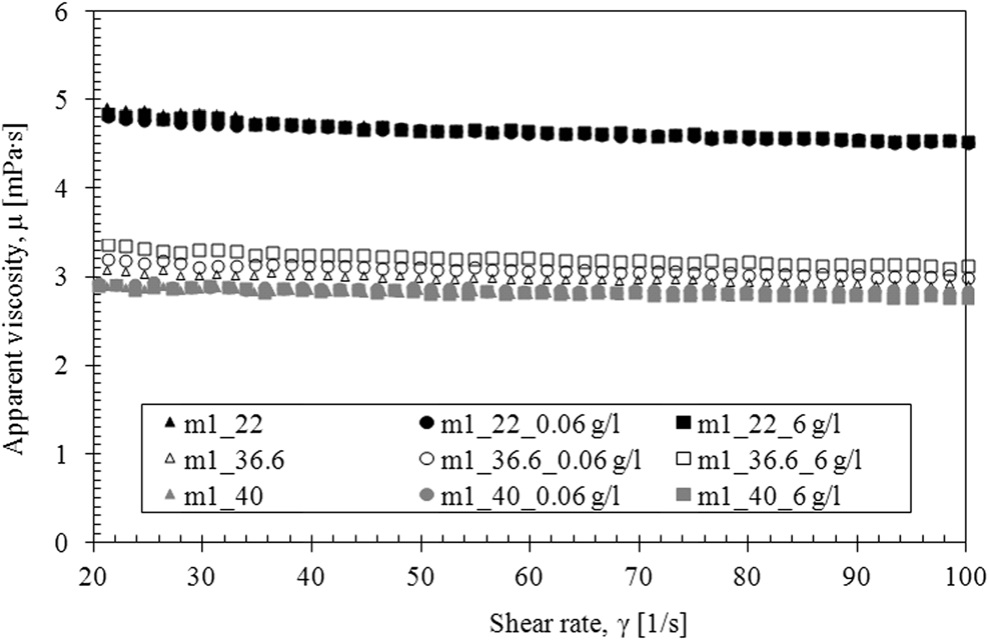
The apparent viscosity of TDDP suspension in mucus model 1

**Fig. 12:**
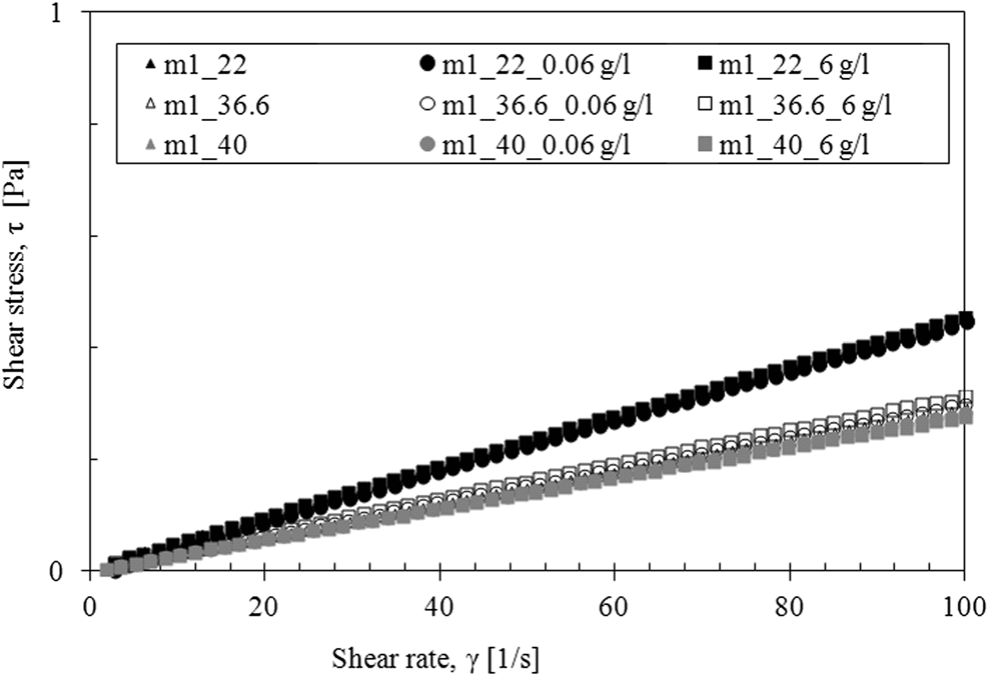
The flow curves for TDDP suspension in mucus model 1

**Fig. 13:**
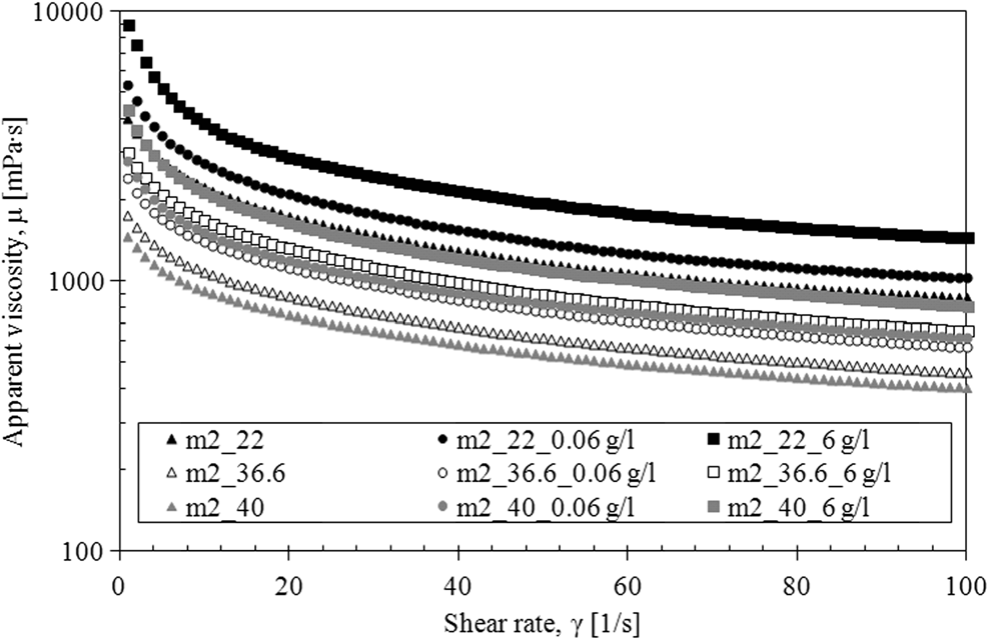
The apparent viscosity of TDDP suspension in mucus model 2

**Fig. 14:**
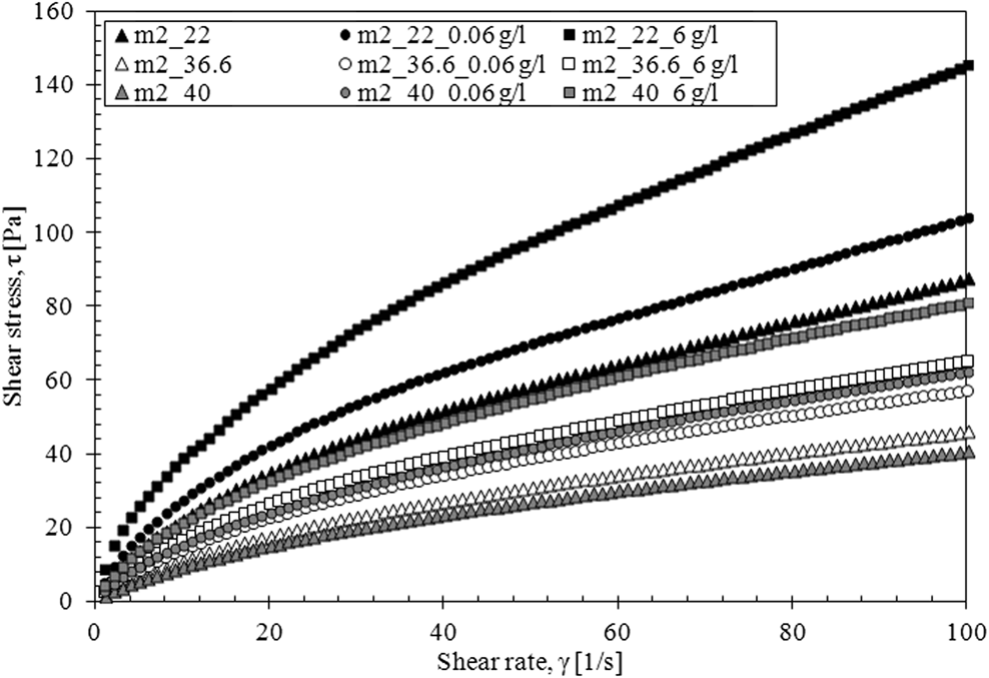
The flow curves for TDDP suspension in mucus model 2

The presence of TDDP in mucus model 1 caused a slight increase in the apparent viscosity at the body temperature (36.6 °C) but had no effect at other temperatures (Fig. 11). The shape of flow curves does not change that indicates that the Newtonian character of fluid is preserved (Fig. 12). The influence of TDDP on rheological properties of mucus model 2 is more pronounced. The apparent viscosity increases while the amount of TDDP in mucus increases at all three investigated temperatures (Fig. 13). The addition of particles does not change the shape of the flow curves; the TDDP suspension in mucus model 2 is still a pseudoplastic fluid (Fig. 14).

**Fig. 15:**
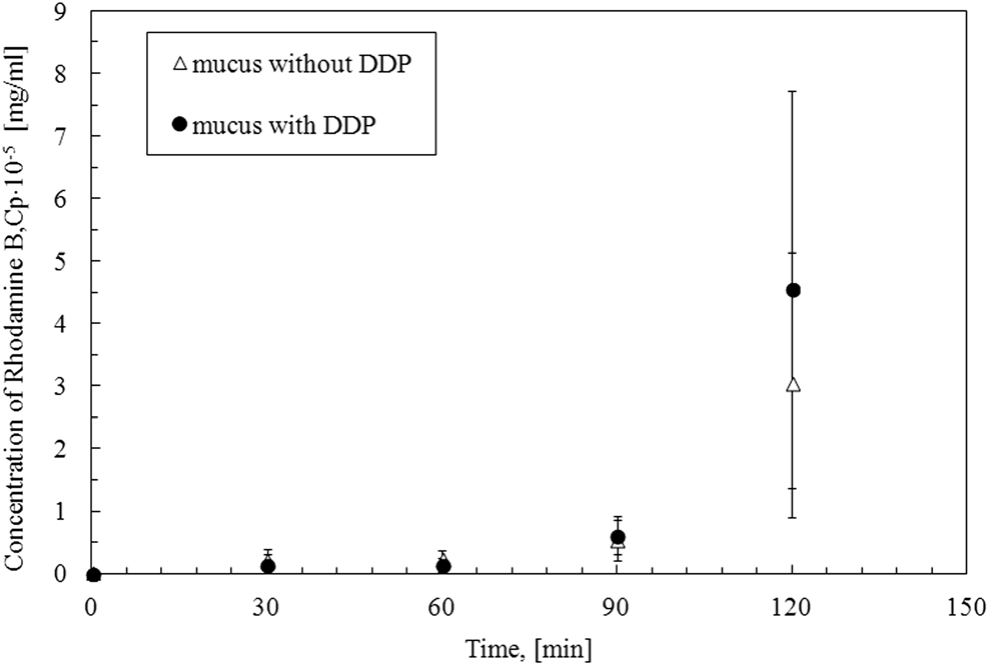
The concentration of rhodamine B in the acceptor side of the diffusion cell when native mucus and mucus with TDDP (mucus with TDDP) are investigated

Because the effect of the presence of TDDP on mucus rheology is more visible for mucus model 2, therefore, this model was used in a further study to investigate the protective properties of mucus contaminated by desert dust particles. The diffusion time of rhodamine B through mucus model 2 contaminated by TDDP (6 g/l) was determined and compared to the time of diffusion through native mucus model 2. The obtained results are shown in Fig. 15.

In both examined cases, the rhodamine B molecules need 120 min to diffuse through mucus regardless of presence or absence of TDDP in mucus. The average amount of rhodamine B diffused through mucus with TDDP is higher than through native mucus; however, due to the very high standard deviations, the results have only an informational rather than a quantitative value. Moreover, taking into account the concentration of rhodamine B after 120 min in the acceptor side and the concentration of rhodamine B in the donor side, it should be noticed that the amount in rhodamine B in the acceptor part is 0.06 and 0.09‰ of the amount in the donor portion for native mucus and mucus with TDDP respectively. The low amount of marker and its long diffusion time allows assuming that the mucus contaminated by TDDP does not lose its protective function. But the increase in mucus viscosity may cause problems with its passage to the upper respiratory tracts and its expectoration, and as a consequence, the retention of mucus in the airways may lead to a general feeling of discomfort or local inflammation.

## Conclusions

The presence of TDDP in saliva and mucus model increased their apparent viscosity. The higher the amount of mucins in the fluid, the higher the observed increase in viscosity. This may lead to the conclusion that the relationship between exogenous particle and mucin chain plays the primary role in the process. Thus, if the biological fluid is naturally more viscous, as a result of, e.g. disease, the impact of TDDP will be greater. Moreover, a higher concentration of particles has a more significant effect.

However, an ambiguous effect of TDDP on saliva and mucus model at different temperatures is puzzling. The impact of the presence of TDDP in fluids on their viscosity should be higher in lower temperature, but such a result was not observed for mucus with a high amount of mucins. The influence of TDDP on mucus at 40 °C is higher than at 36.6 °C but lower than at 22 °C. Indeed, the impact of TDDP on mucus and saliva in various temperatures needs further study.

The TDDP presence in mucus and saliva increases their apparent viscosity that may lead to disturbed moisturising function and difficulty in swallowing. Our results showed that the presence of TDDP with a concentration of 6 g/l in mucus had no significant effects on the diffusion of the fluorescent marker through mucus layer, which may lead to the conclusion that the protective function of mucus would not be disturbed.
